# Ultrasonographic features of gastrointestinal ulcerations in cats

**DOI:** 10.1002/vetr.5222

**Published:** 2025-03-10

**Authors:** Ana Bach, Blanca Serra Gomez de la Serna, Thomas Maddox, Philippa Weston

**Affiliations:** ^1^ Royal Veterinary College Hatfield UK; ^2^ Institute of Infection Veterinary and Ecological Sciences University of Liverpool Neston UK

## Abstract

**Background:**

Gastrointestinal ulceration in cats can be life threatening due to the risk of perforation and septic peritonitis. However, the ultrasound findings associated with this condition and their diagnostic sensitivities have not been described. Therefore, this multicentre retrospective study aimed to describe the clinical features and ultrasound findings for cats with gastrointestinal ulceration and estimate the diagnostic sensitivity of in these cases.

**Methods:**

Hospital medical record databases were retrospectively searched for feline cases with ‘ulcer’ keywords. Cats were included in the study if they had undergone an abdominal ultrasound followed by surgical, endoscopic or postmortem histopathological verification of gastrointestinal ulceration.

**Results:**

Twenty‐four cats were included. On ultrasound examination, all cases showed a mucosal defect filled with hyperechoic microbubbles located in the stomach (29.2%), pylorus (16.7%), duodenum (29.2%), jejunum (20.8%) or ileocecocolic junction (4.2%). Single lesions were present in 75% of cases. Perforations occurred in 16.7% of cases. Wall thickening was detected in 62.5% of the cats, and loss of wall layering was observed in 54.2%. Underlying aetiologies included neoplasia (33.0%), inflammation (33.0%), trauma (12.5%) and foreign bodies (12.5%).

**Limitations:**

The retrospective design limits standardisation of ultrasound techniques and records, thereby potentially limiting the generalisability of the findings.

**Conclusion:**

Ulceration was identified by ultrasound in 75% of cats. Solitary ulcerative lesions with associated wall thickening and crater‐like defects were most commonly documented.

## INTRODUCTION

Gastrointestinal ulceration can be defined as a mucosal defect that exposes the underlying layers of the gastrointestinal wall to gastrointestinal contents.^1^ Gastrointestinal ulceration results from any condition that compromises the normal physiological mechanisms of the gastrointestinal mucosa, such as impaired gastrointestinal wall perfusion, hypersecretion of gastric acid or disruption of the mucus‒bicarbonate barrier.^2^, ^3^ Reported causes of gastrointestinal ulceration in cats include neoplasia, with lymphoma being over‐represented,^4^, ^5^ and less commonly, non‐steroidal anti‐inflammatory drug administration,^5^, ^6^ corticosteroid administration,^4^ inflammatory bowel disease,^5^, ^7^ hypereosinophilic syndrome,^8^, ^9^, ^10^ feline gastrointestinal eosinophilic sclerosing fibroplasia,^11^ endoparasites,^12^, ^13^ infection with *Actinomyces* species^14^ and intoxication.^15^

Cats with gastrointestinal ulceration frequently present in a critical condition.^4^ Life‐threatening sequelae, including gastrointestinal haemorrhage, hypovolaemia and septic peritonitis due to gastrointestinal perforation, are frequently reported.^4^, ^5^ Survival rates associated with gastrointestinal perforation are significantly lower in cats (14%) than in dogs (63%), emphasising the need for earlier detection of ulcerative lesions in cats.^16^

Feline gastrointestinal ulceration has historically been reported as a rare condition; however, a recent study of 61 cats undergoing endoscopy suggested a higher prevalence (5.1%).^5^ This finding may reflect challenges in the diagnosis of feline gastrointestinal ulceration, such as the stoic nature of feline patients, haemorrhage obscuring ulcerative lesions on endoscopy and mesenteric walling‐off of lesions obscuring surgical visualisation. Alternatively, it may be attributed to the unknown diagnostic utility of imaging modalities for the diagnosis of feline gastrointestinal ulcers.^4^

The diagnosis of gastrointestinal ulcers is frequently made by diagnostic imaging, endoscopy or surgery.^4^, ^5^ Although endoscopy is considered the gold standard for the diagnosis of gastrointestinal ulceration, abdominal ultrasound still plays a vital diagnostic role due to its greater accessibility, non‐invasive nature and ability to evaluate other extra‐intestinal structures.^5^, ^17^ The typical ultrasonographic appearance of canine gastrointestinal ulcers is a focal area of mucosal thickening with a central, crater‐shaped mucosal defect containing accumulations of hyperechoic microbubbles.^18^ The sensitivities of radiography, ultrasound and computed tomography for diagnosing gastrointestinal ulcers have been described in dogs, with ultrasound sensitivities of 65% for non‐perforated ulcers and 86% for perforated ulcers,^19^ although other studies report much lower sensitivities for non‐perforated ulcers.^20^ This discrepancy can be explained by the susceptibility of ultrasound to factors such as operator skill, machine settings and patient conformation.^21^ For this reason, extrapolation of published sensitivities in dogs may not accurately represent those in cats, especially due to the anatomical and conformational differences between the two species. To the authors’ knowledge, there are no studies assessing the sensitivity of abdominal ultrasound for diagnosing gastrointestinal ulceration in cats or describing the ultrasonographic features across a larger patient cohort.

The aims of this retrospective, multicentre study were to (1) describe the ultrasonographic appearance of gastrointestinal ulceration in a larger population of cats, (2) ascertain the sensitivity of abdominal ultrasonography for the diagnosis of gastrointestinal ulceration in referral populations of cats, and (3) describe the clinical features of cats diagnosed with gastrointestinal ulceration, including bodyweight, breed, sex, age, location of the gastrointestinal ulcer and the final working diagnosis given. The authors hypothesised that ultrasound would demonstrate good sensitivity, potentially as high or higher than in dogs, due to feline patient conformation.

## MATERIALS AND METHODS

The electronic medical record databases (including patient records and ultrasound, endoscopy, surgical and necropsy reports) of the University of Liverpool Small Animal Teaching Hospital and the Royal Veterinary College's Queen Mother Hospital for Animals were retrospectively searched for feline cases presented between January 2013 and June 2023 using the keywords ‘ulcer’ and ‘ulceration’. Cats were included if they had an abdominal ultrasound performed at the referral hospital, followed by confirmation of gastrointestinal ulceration through endoscopy, surgery or postmortem examination within 48 hours of the ultrasound examination. Cats were excluded if the recorded ulcerative lesion was not within the gastrointestinal tract, if records were incomplete or if the keyword was detected only in the ultrasound report or a confirmatory procedure.

### Technical parameters

Across the two centres, two different ultrasound machines, Logiq 7 (General Electric Medical System) and RS80A system (Samsung Medison), were used, using a linear or microconvex probe with frequencies ranging from 4 to 18 MHz. Patients were positioned in lateral recumbency for scanning. Ultrasonography was performed with the cats restrained under sedation or general anaesthesia. Variable sedative and anaesthetic protocols were selected by the consulting anaesthetist at the time of scanning. All ultrasound examinations were performed by a European College of Veterinary Diagnostic Imaging (ECVDI) board‐certified radiologist or a resident in training under the supervision of the board‐certified radiologist. Endoscopies, exploratory laparotomies and postmortem examinations were performed by a board‐certified internal medicine specialist, surgeon or pathologist, respectively, or a resident under their direct supervision.

Patient bodyweight, breed, sex and age were extracted from the medical records, and clinical history, final working diagnosis and history of previous ulcerogenic drug administration were extracted from referral letters. As previously described, gastrointestinal ulcers were defined as mucosal defects containing hyperechoic microbubbles along the mucosal surface.^18^ Ultrasound features recorded included: the presence and number of gastrointestinal ulcers, the location of the ulcer (pylorus or pyloric antrum, gastric fundus, lesser or greater curvature of the stomach, duodenum, jejunum, ileum, caecum or colon), focal or generalised wall thickening (yes/no), loss of layering (yes/no), echogenicity of the surrounding fat, presence of associated mass lesions (yes/no) and presence of gastrointestinal distention or ileus (yes/no). Each study was recorded as positive if gastric and/or enteric ulceration was specifically documented at the conclusion of the imaging report. Where identified, additional ultrasonographic features recorded included the presence of an associated gastrointestinal foreign body, evidence of pancreatic inflammation, abdominal lymphadenopathy and evidence of free abdominal fluid or gas. Ulcer presence was confirmed with either endoscopy, exploratory laparotomy, gastroenterotomy or postmortem findings. Where relevant and available, histopathology results and follow‐up information were reviewed. For all methods of diagnostic confirmation, the presence and location of ulcerative lesions were documented.

The data were entered into a spreadsheet (Microsoft Excel; Microsoft Corporation) and summary statistics were generated. Sensitivity was calculated in Microsoft Excel by an ECVDI diagnostic imaging resident (A.B.).

## RESULTS

### Population

**FIGURE 1:**
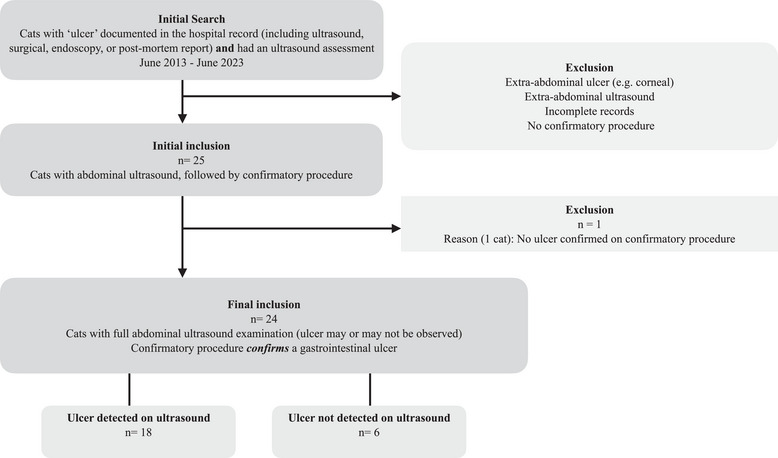
Patient inclusion flow chart

Twenty‐four cats met the inclusion criteria (Figure 1). Details for individual cases are provided in the Supporting Information. The included cats had a median age of 7.5 years (range 1‒15 years). The breeds included 19 domestic short hair, three British short hair, one Persian, one Siamese, one Ragdoll and one British Blue. There were 10 female neutered cats, three male entire cats and 12 male neutered cats. Bodyweight was recorded for 16 patients, with a median weight of 4.0 kg (range 3–6.3 kg).

### Clinical presentation

Of the 24 cases, 14 (56.0%) were acute cases (defined as having clinical signs of less than 3 weeks' duration) and 10 (41.6%) were chronic. Patients presented with vomiting (15/24, 62.5%), lethargy (9/24, 37.5%), hyporexia (8/24, 33.3%), anorexia (6/24, 25.0%), weight loss (5/24, 20.8%), melena (3/24, 12.5%), anaemia (1/24, 4.2%), ptyalism (1/24, 4.2%), constipation (1/24, 4.2%), collapse (1/24, 4.2%), haematemesis (1/24, 4.2%) or following trauma (2/24, 8.4%).

### Detection of ulcers

Ultrasound diagnosis of an ulcer was made in 18 (75%) cases. However, ultrasound did not detect ulcerations in six cats (25%), which were confirmed as false negatives based on subsequent diagnostic procedures. Ulceration was confirmed by endoscopy in 10 cats (42%), exploratory laparotomy in 12 cats (50%) and postmortem examination in two cats (8%). The calculated sensitivity for the detection of ulceration by ultrasound was 75% (95% confidence interval 55–88%).

### Imaging features

On ultrasound, all cats demonstrated either crater‐like or irregular defects along the luminal mucosal surface, containing focal regions of hyperechogenicity with reverberation artefact. These were located at the stomach (7/24, 29.2%), pylorus or pyloric antrum (4/24, 16.7%), proximal duodenum (6/24, 25.0%), jejunum (5/24, 20.8%), middle to distal duodenum (1/24, 4.0%) or ileocecocolic junction (1/24, 4.2%). In all cases, the location of gastrointestinal ulcers identified on ultrasound corresponded with the findings of confirmatory diagnostic methods. In one instance, the surgical report was more precise, describing the ulcer as located in the lesser curvature, whereas the ultrasound report noted the gastric body. For the purposes of this study, this case was considered an agreement. Seventy‐five percent of cases had single lesions. Focal or generalised wall thickening was detected in 15 (62.5%) cats, while loss of wall layering was reported in 14 (58.3%), presence of gastric foreign body in three (12.5%) and gastric pneumatosis in one (4.2%). Mass‐like lesions were identified in 13 (54.2%) cases.

Associated abdominal changes (i.e., tertiary changes) included locoregional abdominal lymphadenomegaly in eight cats (33.3%), mesenteric hyperechogenicity in seven (29.2%), free peritoneal fluid in six (25.0%) and peritoneal gas in four (16.7%).

**FIGURE 2:**
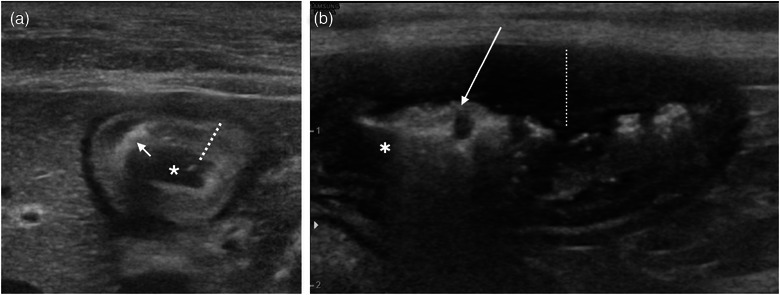
Examples of the ultrasonographic appearance of non‐neoplastic ulceration in the duodenum of two cats. (a) A single ulcer secondary to lymphoplasmacytic‒neutrophilic duodenitis in the proximal duodenum. The mucosa (dotted line) is diffusely echogenic and there is a small amount of luminal fluid (*) present. The mucosal surface is focally irregular, with gas tracking into the mucosa to the level of the submucosa (arrow). Layering distinction is preserved. (b) Extensive duodenal ulceration from the mid‐point of the descending duodenum to the caudal duodenal flexure, secondary to suspected trauma. The image shows moderate to marked asymmetric irregularity of the luminal surface of the mucosa. Luminal gas (*) extends intramurally (arrow). There is also mild to moderate eccentric mural thickening (dotted line) up to 6 mm thick, with reduced layering differentiation. The affected region is aborad to the major and minor duodenal papillae

Perforations were suspected in four (16.7%) cases, based on the detection of free peritoneal gas and/or fluid, and were later confirmed by surgery or postmortem. In one case, the single perforating ulcer site was directly visualised on ultrasound. In the second case, multiple gastric ulcers were observed by ultrasound and confirmed postmortem, but the perforating lesion was not described. This was specified to be at the pylorus on postmortem. These two cases were classified as detected on ultrasound. In the remaining two perforated cases, an ulcer was not directly observed on ultrasound, but secondary signs of perforation (pneumoperitoneum and peritoneal effusion) prompted suspicion of perforation, which was subsequently confirmed during surgery. These two cases were classified as not detected on ultrasound. The causes of the perforated ulcers were trauma (2/4, 50.0%), a foreign body (1/4, 25.0%) and non‐steroidal anti‐inflammatory medication (1/4, 25.0%). In one case, the perforation site was identified ultrasonographically as hyperechoic gas foci traversing from the mucosal to the serosal layer (Figure 2). On surgical or postmortem assessment, perforations were located at the proximal duodenum (2/4, 50.0%), pylorus (2/4, 50.0%) or lesser curvature of the stomach (1/4, 25.0%).

### Aetiologies

**FIGURE 3:**
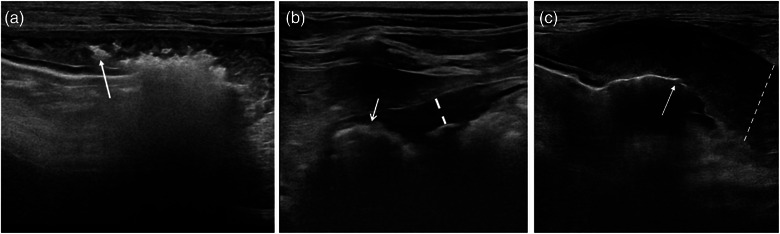
Ultrasonographic images of gastric lymphoma in three cats. (a) Shows gastric pneumatosis (arrow) with multiple intramural gas bubbles in the gastric body. The entirety of the gastric muscular layer is abnormally heterogenous and thickened (up to 7 mm). (b) Shows the antral portion of the stomach with evidence of gas penetrating almost to the serosal surface (arrow). The adjacent gastric wall is thickened (dotted line) and has loss of distinct wall layering. (c) Shows a crater‐like defect (arrow) at the gastric fundus, within a markedly thickened gastric wall (dotted line) with loss of layering. At the left periphery of the image, a transition to a normal mural thickness and more distinct layering is present

The underlying aetiologies of the ulcerations in the study population included neoplasia ([8/24, 33%], of which lymphoma [Figure 3] was most common [6/8, 75%], followed by carcinoma [1/8, 12.5%] and leiomyosarcoma [1/8, 12.5%]), inflammation (8/24, 33%), trauma (3/24, 12.5%) and foreign bodies ([3/24, 12.5%], of which trichobezoars [2/3, 66%] were the most common). Prescribed ulcerogenic treatment included non‐steroidal anti‐inflammatory medication (6/24, 25%) and corticosteroids (5/24, 21%), of which two (2/24, 8.4%) cats received both.

## DISCUSSION

In this study, ulceration was identified by ultrasound in 75% of cats, which is reasonable sensitivity for this small patient cohort. However, this sensitivity reflects the inherent inclusion bias of the study, as all included cases were selected based on confirmation of ulceration by gold‐standard diagnostic methods.

The sensitivity reported herein is higher than in dogs, where sensitivities of 29.5‒65% for non‐perforated ulcers and 86% for perforated ulcers have been documented.^19^, ^20^ The smaller feline body conformation and lower average bodyweight permit the use of higher frequency linear ultrasound probes in the majority of feline abdominal ultrasounds.^17^ The resultant increase in respective axial and lateral resolution may contribute to the higher sensitivity of ultrasound for detecting gastrointestinal ulcers in cats.^21^ Another contributor may be that lesions are often more advanced at the point of diagnostic imaging in cats, meaning that the size and extent of ulceration are less subtle than in canine patients.^4^ Similarly, the prevalence of primary lesions such as neoplasia (50%) and foreign bodies (12.5%) was higher in this feline population compared to studies in canine patients, which may have aided detection of ulceration. Lastly, both non‐perforated and perforated ulcers were included in this study, which has previously been shown to increase the sensitivity of ultrasound from 65% to 86% in canine patients.^19^ This may be due to the higher prevalence of secondary features such as hyperechoic mesenteric fat and presence of peritoneal fluid in cases with perforated ulcers, even in the absence of pneumoperitoneum.^22^

False‐negative results, where ulcers were present but not detected on ultrasound, may arise due to the inherent challenges of gastrointestinal ultrasonography, including the presence of gas artefact and overlying anatomy that can obscure mucosal defects. Additionally, misinterpretation of subtle findings cannot be entirely excluded. These challenges reflect the technical limitations of ultrasonography, for example, physiological variations, machine settings and operator skill.^21^

In agreement with previous ultrasonographic descriptions of gastrointestinal ulceration, all cases in this study had crater‐like or irregular mucosal defects. They were commonly associated with either focal or generalised wall thickening (62.5%), loss of layering (58.3%) and a hyperechoic mesentery (29%), similar to previous reports in dogs.^18^, ^19^, ^20^ Lesions were more commonly single in nature (19/24, 79%); however, multiple ulcerations were found in five of the 24 (21%) cats, all of which were benign in aetiology. The prevalence of multiple lesions in this study is greater than previously reported in canine patients but concurs with a previous endoscopic study where 19 of 62 (31%) cats had multiple ulcerative lesions.^5^

In contrast to previous studies, the majority (13/24, 54.4%) of lesions were enteric in location, mostly duodenal (7/24, 29.1%) or jejunal (5/24, 20.8%). Gastric, particularly antropyloric, ulcerations are historically more commonly reported in cats, with a prevalence of 56% in previous studies.^5^ This is likely reflective of differences in study design, as previous studies either only report gastroduodenal ulceration^4^, ^5^ or are based on endoscopic findings, meaning that detection of jejunal ulceration may be lower due to the physical limitations of some endoscopes.^5^ We report ulceration of the ileocecocolic junction in one case of alimentary lymphoma. However, no caecal ulcerations were reported in this study, emphasising the rarity of caecal ulceration in cats, with only one case of spontaneous caecal perforation due to non‐neoplastic ulceration being previously reported.^23^ Histopathology of this case confirmed transmural enteritis; however, the presence of diffuse infiltrative enteritis was not established.^23^ Nevertheless, the findings of this case report, together with the present study, support a thorough investigation of the caecum and ileocecocolic junction, which are common sites of lymphoma and immune‐mediated or infectious inflammation in cats.^23^, ^24^

Neoplasia accounted for one‐third (8/24) of cases in our study, with leiomyosarcoma accounting for a larger number (12.5%) of cases than previously reported. This prevalence of neoplasia is slightly lower than that previously reported, perhaps due to the lower average age of this study group compared to previous studies (median of 7.6 years, compared to 9 years) or increased representation of non‐neoplastic aetiologies. Neoplasia is a common cause of gastrointestinal rupture in cats, accounting for 54.5% (6/11) of gastrointestinal ruptures in one study.^25^ In the current study, one case with infiltrative lymphoma had associated gastric pneumatosis, an infrequently reported sequelae to neoplasia/neoplastic ulceration in cats.^26^, ^27^ However, neoplasia did not cause perforation in any of our cases, likely due to our inclusion criteria, as cats with perforated gastrointestinal neoplasia are unlikely candidates for surgery or endoscopy.

The majority (66%, 16/24) of lesions in this study were non‐neoplastic. One‐third (8/24) of cases had confirmed underlying inflammatory disease. Several types of inflammatory bowel disease exist in cats, of which the most common are chronic enteropathy, lymphocytic‒plasmacytic enteritis and eosinophilic gastroenteritis.^28^ Also reported, but less commonly, are neutrophilic enteritis and granulomatous enteritis.^29^, ^30^ In our study, five cats were diagnosed with lymphoplasmacytic inflammation affecting the stomach, duodenum and/or jejunum. *Helicobacter* infections are associated with lymphoplasmacytic gastritis in cats, and indeed, *Helicobacter* was determined to be the underlying cause in two cases of lymphoplasmacytic gastritis from our cohort.^31^ Another case had a combination inflammatory condition with both lymphoid and neutrophilic components. Neutrophilic inflammation has been associated with *Campylobacter* infection in cats, but this was not isolated in the case in our cohort.^29^ A surprising number (33.3%, 8/24) of ulcers in our cohort were caused by less commonly reported aetiologies, such as trauma, foreign bodies and trichobezoars. Trichobezoars were present in two of our cases, one gastric and one jejunal. Trichobezoars have been infrequently reported as causes of upper and lower gastrointestinal tract obstruction in cats. A single case of gastrointestinal perforation was reported in a cat with a trichobezoar; however, no ulceration was reported.^22^

This study is limited by its small sample size, multicentre design and retrospective methodology. Despite a wide retrospective search spanning 10 years across two referral institutions, only 24 cats met the inclusion criteria. This small sample size may reflect the low prevalence of feline gastrointestinal ulcers, which are historically cited as a rare condition in cats.^4^ However, a prevalence of 5.1% in cats undergoing endoscopy within a referral population was recently reported, suggesting that these lesions are underdiagnosed in cats.^5^ Due to the retrospective collection of data across multiple institutions, there is variability in the use of ultrasound machinery and operator experience. This study is further limited by the lack of histopathology in several cases; hence, the presence or absence of neoplasia cannot be fully confirmed in all patients. Future prospective studies with histopathological assessment of all cases are therefore merited.

In conclusion, ultrasound is a sensitive tool for detecting gastrointestinal ulcerations in cats. Most commonly, a single mucosal defect with thickening and loss of layering was observed. Gastric lymphoma was a common underlying diagnosis. Abdominal changes in the absence of a conspicuous ulcerative lesion should increase the examiner's confidence in suspicion of a gastrointestinal ulcer.

## AUTHOR CONTRIBUTIONS

Philippa Weston conceived the presented idea. Ana Bach and Blanca Serra Gomez de la Serna collected data across respective centres. Ana Bach and Philippa Weston wrote and reviewed the manuscript. Philippa Weston and Blanca Serra Gomez de la Serna reviewed the ultrasound images. Thomas Maddox reviewed the manuscript and assisted with the data analysis. All the authors discussed the results and contributed to the final manuscript.

## CONFLICT OF INTEREST STATEMENT

The authors declare they have no conflicts of interest.

## FUNDING INFORMATION

This research received no specific grant from any funding agency in the public, commercial, or not‐for‐profit sectors.

## ETHICS STATEMENT

Ethical approval (ethical approval number: VREC1446) for this study was granted by the University of Liverpool's Veterinary Research and Ethics Committee, and in accordance with the ethical guidelines of the Royal Veterinary Colleges.

## Supporting information



Supporting Information

## Data Availability

The data that support the findings of this study are available from the corresponding author upon reasonable request.
