# Supplementary material for: Ultrasonographic features of gastrointestinal ulcerations in cats
Source: Vet Rec. 2025 Mar 10;197(11):e282–7. doi: 10.1002/vetr.5222 (PMC12661621; doi:10.1002/vetr.5222)
Supplement: Supplementary file 1 — Supporting Information [file VETR-197--s001.pdf]

Case	Signalment	Duration of clinical signs	Clinical Signs	Ulcerogenic Drug Administration	Aetiology	Ulcer diagnosed on ultrasound	Location	Number (single /multiple)	Ultrasound findings	Perforation	Confirmation	Correctly diagnosed	Outcome
1	15y FN DSH	4 months	Chronic vomiting, diarrhoea	-	Lymphoid gastroduodenitis	-	Stomach	Single	Ulcer not found	-	Endoscopy		Lost to follow up
2	13y FN BSH	3 months	Chronic vomiting	-	Lymphocytic-plasmocytic/ follicular gastritis. Helicobacter.	Yes	Fundus and lesser curvature	Single	Focal thickening and crater-like defect	-	Endoscopy	Yes	Lost to follow up
3	4y MN DSH	1 week	Anorexia, ptyalism, ileus, lethargy	NSAID	Gastric trichobezoar.	Yes	Lesser curvature	Single	Focal thickening and crater-like defect	Yes	Surgery	Yes	Lost to follow up
4	8y MN Persian	3 months	Chronic regurgitation, vomiting	-	Follicular duodenitis, Helicobacter	-	Proximal duodenum	Multiple	Marked mural thickening, undulating mucosal surface, ulcer not found	-	Endoscopy	-	Repeat endoscopic biopsy after 3 years confirmed inflammatory bowel disease, and relapse in duodenal ulcer which responded to medical treatment
5	1y FN Siamese	5 days	Road traffic accident, then hypersalivation during hospitalisation	-	Trauma: Dog attack	-	Proximal duodenum	Single	Focal mural thinning with hyperechoic surface, ulcer not found	Yes	Surgery	-	Cardiac arrest 2 days following surgery
6	8y FN DSH	8 months	Chronic vomiting, weight loss	Steroid	Duodenitis, bacterial overgrowth.	-	Proximal duodenum	Single	Generalised mural thickening, ulcer not found	-	Surgery	-	Euthanasia after an episode of weakness and hematochezia 4 months later
7	9y ME DSH	2 months	Vomiting, anorexia, weight loss	-	Duodenal carcinoma	Yes	Proximal duodenum	Single	Focal thickening and crater-like defect	-	Surgery	Yes	Cardiac arrest 4 days following surgery
8	6y FN Ragdoll	4 days	Refusing to move, progressive lethargy and hyporexia	NSAID	Gastric lymphoma	Yes	Antrum and body	Multiple	Generalised mural thickening, pneumatosis	-	Post-mortem	Yes	Euthanasia
9	10y FN DSH	6 months	Chronic vomiting and hyporexia	-	Gastric lymphoma	Yes	Fundus	Single	Focal thickening and crater-like defect	-	Endoscopy	Yes	Palliative treatment
10	7y MN DSH	1 month	Inappetence, anorexia and chronic intermittent constipation	-	Gastric lymphoma	-	Pyloric antrum	Single	Mass lesion with adjacent mucosal irregularity	-	Endoscopy	-	Lost to follow up
11	9y MN DSH	2 weeks	Anorexia, vomiting, hematemesis, lethargy	-	Gastric lymphoma	Yes	Stomach	Single	Focal thickening and crater-like defect	-	Endoscopy	Yes	Lost to follow up
12	4y MN DSH	6 weeks	Vomiting	-	Gastric lymphoma	Yes	Pyloric antrum	Single	Focal thickening and crater-like defect	-	Endoscopy	Yes	Lost to follow up
13	4y ME BSH	1 week	Vomiting, melena (3 days prior), then road traffic accident	NSAID, Steroid	Trauma; road traffic accident	-	Pylorus	Single	Pneumoperitoneum and effusion, ulcer not found	Yes	Surgery		Lost to follow up
14	7y FN DSH	4 weeks	hyporexia, lethargy, previously 1 year history of intermittent vomiting and diarrhoea	-	Lymphocytic- neutrophilic gastritis	Yes	Gastric	Single	Diffuse thickening, multiple mucosal hyperechoic foci	-	Endoscopy	Yes	Lost to follow up
15	7y MN DSH	3 weeks	Hyporexia, lethargy, weight loss, pyrexia	-	Alimentary lymphoma	Yes	ICCJ	Single	Circumferential thickening, crater-like defect	-	Surgery	Yes	Lost to follow up
16	4y MN DSH	2 weeks	Hyporexia, lethargy, weight loss, pyrexia	NSAID, steroid	Fibronecrotic jejunitis	Yes	Jejunum	Single	Irregular hyper echoic mucosal defect	-	Surgery	Yes	Lost to follow up
17	8y FN DSH	1 week	Anorexia, vomiting, lethargy	NSAID	Lymphoplasmacytic jejunitis	Yes	Jejunum	Multiple	Focal thickening, crater-like defect, mural gas-trapping	-	Surgery	Yes	Lost to follow up
18	12y FN DSH	2 weeks	Hyporexia, vomiting	-	Trauma	Yes	Duodenum	Single	Focal thickening, mucosal irregularity	-	Surgery	Yes	Lost to follow up
19	12y MN DSH	1 day	Lethargy, collapse	-	Ulcerative jejunitis, trichobezoars	Yes	Jejunum	Single	Focal hyperechoic region within mucosa	-	Surgery	Yes	Lost to follow up
20	8y MN DSH	1 week	Hyporexia, vomiting, inappropriate urination	NSAID	Presumed NSAID use	Yes	Pylorus	Multiple	Multiple areas of thickening, intramural gas bubbles, pneumoperitoneum	Yes	Post-mortem	Yes	Euthanasia
21	6y MN DSH	1 week	Hyporexia, lethargy, panting, melena, one-off vomiting	-	Jejunal leiomyosarcoma	Yes	Jejunum	Single	Focal thickening, loss of layering, intramural hyperechoic foci	-	Surgery	Yes	Repeat ultrasound 19 months after surgery (no recurrence, clinically well)
22	5y MN BSH	16 weeks	Vomiting, weight loss, dermatitis (pruritus and flaky skin)	Steroid	Lymphoplasmacytic - neutrophilic duodenitis	Yes	Proximal duodenum	Single	Focally irregular mucosa, gas-tracking	-	Endoscopy	Yes	Lost to follow up
23	13y MN British Blue	1 day	Melena and anaemia	-	Hyperplastic Duodenal polyp	Yes	Proximal duodenum	Multiple	Multiple, ill-defined intralesional hyperechoic foci	-	Endoscopy	Yes	Lost to follow up
24	1y FN DSH	1 week	Vomiting, lethargy	-	Jejunal foreign body	Yes	Jejunum	Single	Crater-like defect, intraluminal content with distal acoustic shadowing	-	Surgery	Yes	Lost to follow up
